# Recurrent CSF Rhinorrhea Misdiagnosed as Chronic Allergic Rhinitis with Subsequent Development of Bacterial Meningitis

**DOI:** 10.1155/2017/9012579

**Published:** 2017-07-26

**Authors:** Michael T. Ulrich, Lawrence K. Loo, Michael B. Ing

**Affiliations:** ^1^Department of Medicine, Loma Linda University Medical Center, Loma Linda, CA, USA; ^2^Infectious Diseases Section, Jerry L. Pettis Memorial Veterans Hospital, Loma Linda, CA, USA

## Abstract

**Introduction:**

Cerebrospinal fluid (CSF) rhinorrhea results from an abnormal communication of the dura mater to the nasal mucosa. The majority of cases of CSF rhinorrhea are the result of trauma or surgery involving the skull base. Spontaneous CSF rhinorrhea is a rare clinical entity with increased risk of ascending infection. Delay in diagnosis places the patient at risk of developing meningitis.

**Case Presentation:**

A 36-year-old African American female with significant medical history of obesity and hypertension presented to the emergency department with headache, altered level of consciousness, fever, and neck stiffness. Previously, the patient was diagnosed with chronic allergic sinusitis by multiple providers. Physical exam findings and laboratory tests were consistent with bacterial meningitis. The patient was admitted and started on appropriate antibiotic therapy. The patient continued to complain of persistent unilateral clear nasal drainage. The initial report from the computerized tomography scan of the sinuses indicated findings consistent with chronic sinusitis. Magnetic resonance imaging of the orbits revealed findings consistent with CSF rhinorrhea. Otolaryngology was consulted for surgical intervention.

**Conclusion:**

Suspected CSF rhinorrhea should prompt immediate biochemical and radiologic evaluation and surgical consultation. CSF rhinorrhea places patients at risk of developing bacterial meningitis.

## 1. Introduction

Cerebrospinal fluid (CSF) rhinorrhea results from an abnormal communication of the dura mater to the nasal mucosa. The majority of cases of CSF rhinorrhea are the result of trauma or surgery involving the skull base; however, spontaneous CSF rhinorrhea is a rare clinical entity. This syndrome has potentially life-threatening sequela, and a delay in diagnosis places the patient at risk of developing bacterial meningitis with a mortality rate of approximately 14% [1]. The organisms that lead to an ascending meningitis or meningoencephalitis are typically flora from the upper respiratory tract.* Streptococcus pneumoniae* is the most common organism and has been implicated in recurrent bacterial meningitis in this patient population [2]. There are both invasive and noninvasive methods for the detection of CSF rhinorrhea and their diagnostic utility has been well documented. CSF rhinorrhea must be included in the differential diagnosis for patients with persistent nasal discharge or the diagnosis may be delayed. Below we present one such case.

## 2. Case Presentation

A 36-year-old African American female with significant medical history of obesity and hypertension presented to the emergency department with headache, altered level of consciousness, fever, and severe neck stiffness. The patient was previously evaluated by multiple providers in the emergency department (urgent care) and was diagnosed with chronic allergic sinusitis. She was prescribed multiple courses of antibiotics with supportive care and sent home. She had recent animal exposure to a stray cat and consumed deli meats regularly. There was no history of recent travel. She denied any mosquito or tick bites.

On physical exam, the patient was febrile, tachycardic, tachypneic, and disoriented to place and time with inappropriate responses to questioning, and nuchal rigidity was present. Complete blood cell count revealed a white blood cell count of 16.88 × 10^9^ per liter with 80% band forms and 14% segmented neutrophils. Comprehensive metabolic panel showed no electrolyte abnormalities or renal or liver dysfunction. Urine analysis was within normal limits. CSF analysis revealed WBC count of 7810 cells/*μ*L with 83% neutrophils, red blood cells count of 22 cells/*μ*L, protein of 267 mg/dL, and glucose of 27 mg/100 mL. Gram stain, West Nile Virus antibody titer, and Coccidioides antibody were negative. Serum lactate was within normal limits. Unfortunately, the patient had received intravenous (IV) antibiotics prior to lumbar puncture and the CSF cultures remained negative. The CSF was not sent for detection of bacterial antigens.

Despite the negative gram stain and cultures of the CSF, the clinical presentation and results of lumbar puncture were consistent with bacterial meningitis. In the emergency department, the patient was started on broad-spectrum antibiotics and antivirals with vancomycin, piperacillin/tazobactam, and acyclovir. On the medicine service, antibiotics and antivirals were transitioned to standard therapy for bacterial meningitis with ceftriaxone and vancomycin. The patient had resolution in her altered level of consciousness by the following day and was able to answer questions.

**Figure 1:**
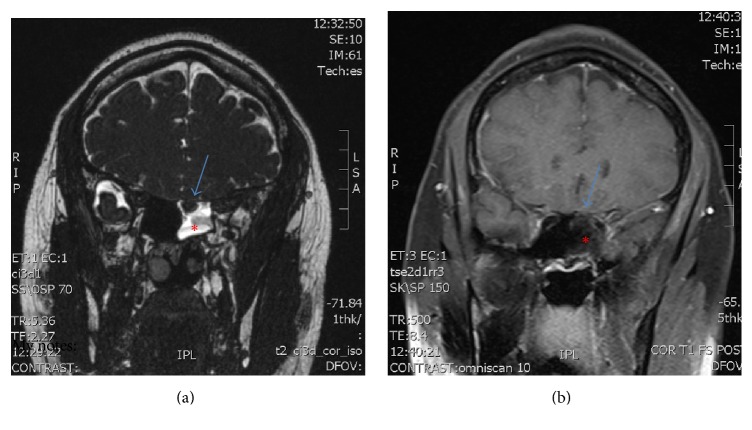
T2 (a) and T1 (b) weighted images demonstrating a basilar skull defect with meningocele (arrow) and fluid accumulation in the left sphenoid sinus (asterisk).

Upon further questioning, she endorsed persistent unilateral clear nasal drainage lasting months and postural headache that was worse when standing or sitting and relieved by laying down. At this time, there was high clinical suspicion of CSF rhinorrhea. Computerized tomography (CT) scan of the sinuses initially indicated findings consistent with chronic sinusitis. However, the patient continued to have copious unilateral clear nasal discharge. Magnetic resonance imaging (MRI) of the orbits was completed which showed findings consistent with CSF rhinorrhea and bony defect of the basal skull (Figure 1). Additionally, her nasal discharge was positive for beta-2 transferrin. Otolaryngology was consulted for further evaluation. The patient was taken to surgery and intraoperative findings were consistent with CSF rhinorrhea of the left sphenoid sinus. The mucosal defect was repaired with free septal mucosal graft and tissue seal. The patient recovered without complications and was discharged home to complete a course of IV antibiotics.

## 3. Discussion

The clinical context of most reported cases of CSF rhinorrhea is that of head trauma, “minor trauma,” or previous surgical intervention. However, spontaneous CSF rhinorrhea is an emerging clinical entity. Investigators have identified several potential risk factors for this syndrome such as elevated body mass index (BMI), obstructive sleep apnea (OSA), and intracranial hypertension [3–6]. Although the exact pathogenesis of spontaneous CSF rhinorrhea is not known, investigators have speculated that defects in the skull base may develop as a result of elevated intracranial pressures [7]. One explanation is that individuals who have a predisposition to this syndrome and develop intracranial hypertension may spontaneously form a dural-mucosal fistula.

Clinical suspicion of spontaneous CSF rhinorrhea should prompt immediate radiologic evaluation and surgical consultation. This syndrome places patients at an increased risk of developing bacterial meningitis, which carries a high mortality rate [8, 9]. This case presents a particular dilemma, as multiple providers evaluated the patient and her diagnosis was delayed. Even with high clinical suspicion of CSF rhinorrhea, the initial CT scan was nondiagnostic. It was only after conformation with MRI findings and analysis of the nasal drainage that the diagnosis was made.

The least invasive confirmatory tests for CSF rhinorrhea are the presence of either beta-2 transferrin or beta-trace protein in the nasal discharge, which are sensitive and specific for CSF and perilymph fluids [10, 11]. There are data to support the use of either high-resolution CT scan, MRI, or CT/MRI cisternography for diagnosis of the basilar skull defects in CSF rhinorrhea [12]. In many clinical settings, it is reasonable to begin with analysis of the nasal discharge and HRCT imaging if available. In cases of high clinical suspicion, where imaging studies are negative, intrathecal fluorescein can be utilized to localize lesions; however, there is an appreciable false-negative rate of approximately 25% [13].

Data extrapolated from patients with basal skull fractures who develop CSF rhinorrhea does not support a role of prophylactic antibiotic therapy in the prevention of meningitis, and no randomized control trial has yet been conducted to evaluate this question [14]. However, CSF rhinorrhea is an indication for vaccination with pneumococcal conjugate vaccine (PCV 13 and PCV 23) [15]. Patients with CSF rhinorrhea can be treated with conservative management, such as elevating the head of the bed and placement of a lumbar drain. Alternatively, these patients may need surgical repair with either open craniotomy or endoscopy. The need for invasive or conservative treatment is guided by the rate of flow from the dural-mucosal fistula, as high flow lesions are not likely to resolve spontaneously. In either context, surgical consultation will help guide management for these patients [16].

## 4. Conclusion

We have the following conclusions:

- Consider the diagnosis of spontaneous CSF rhinorrhea in patients that present with clear sinus drainage, postural headache, and clinical signs of intracranial hypertension.
- Diagnosis is made reliably with positive beta-2 transferrin or beta-trace protein in combination with HRCT or MRI.
- Patients with spontaneous CSF rhinorrhea are at an increased risk of developing bacterial meningitis, but the use of prophylactic antibiotics in CSF rhinorrhea is not supported by randomized control trial and systematic reviews have not revealed benefit.
- CSF rhinorrhea is an indication for PCV vaccination.
